# Autism and Increased Paternal Age Related Changes in Global Levels of Gene Expression Regulation

**DOI:** 10.1371/journal.pone.0016715

**Published:** 2011-02-17

**Authors:** Mark D. Alter, Rutwik Kharkar, Keri E. Ramsey, David W. Craig, Raun D. Melmed, Theresa A. Grebe, R. Curtis Bay, Sharman Ober-Reynolds, Janet Kirwan, Josh J. Jones, J. Blake Turner, Rene Hen, Dietrich A. Stephan

**Affiliations:** 1 Center for Neurobiology and Behavior, Department of Psychiatry, University of Pennsylvania, Philadelphia, Pennsylvania, United States of America; 2 Neurogenomics Division, Translational Genomics Research Institute, Phoenix, Arizona, United States of America; 3 Southwest Autism Research and Resource Center, Phoenix, Arizona, United States of America; 4 Division of Child and Adolescent Psychiatry, Department of Psychiatry, Columbia University, New York, New York, United States of America; 5 Departments of Psychiatry and Neuroscience, Columbia University, New York, New York, United States of America; Brunel University, United Kingdom

## Abstract

A causal role of mutations in multiple general transcription factors in neurodevelopmental disorders including autism suggested that alterations in global levels of gene expression regulation might also relate to disease risk in sporadic cases of autism. This premise can be tested by evaluating for changes in the overall distribution of gene expression levels. For instance, in mice, variability in hippocampal-dependent behaviors was associated with variability in the pattern of the overall distribution of gene expression levels, as assessed by variance in the distribution of gene expression levels in the hippocampus. We hypothesized that a similar change in variance might be found in children with autism. Gene expression microarrays covering greater than 47,000 unique RNA transcripts were done on RNA from peripheral blood lymphocytes (PBL) of children with autism (n = 82) and controls (n = 64). Variance in the distribution of gene expression levels from each microarray was compared between groups of children. Also tested was whether a risk factor for autism, increased paternal age, was associated with variance. A decrease in the variance in the distribution of gene expression levels in PBL was associated with the diagnosis of autism and a risk factor for autism, increased paternal age. Traditional approaches to microarray analysis of gene expression suggested a possible mechanism for decreased variance in gene expression. Gene expression pathways involved in transcriptional regulation were down-regulated in the blood of children with autism and children of older fathers. Thus, results from global and gene specific approaches to studying microarray data were complimentary and supported the hypothesis that alterations at the global level of gene expression regulation are related to autism and increased paternal age. Global regulation of transcription, thus, represents a possible point of convergence for multiple etiologies of autism and other neurodevelopmental disorders.

## Introduction

Autism is a severe neurodevelopmental disorder with characteristic social and communication deficits and ritualistic or repetitive behaviors that appear by age three. Many etiologies have been suggested and numerous risk factors have been identified [1]. Though autism is associated with a high degree of heritability, few specific genetic mutations have been identified accounting for a minority of cases [2], [3], [4], [5], [6], while the majority of cases are considered sporadic. The failure to identify specific gene variants for most cases of autism has been attributed to many potential factors including complex interactions of multiple genes, a heterogeneous disorder with multiple causes converging on the autistic phenotype, or epigenetic factors not related to specific genetic mutations or polymorphisms [2], [3]. None of these hypotheses has been confirmed and they are not mutually exclusive.

Research on gene expression in autism has previously focused on identifying specific or a limited group of genes related to disease [7], [8], [9]. The idea that alterations at the global level of gene expression regulation might be important in mediating the risk for autism or other disease states has been largely underexplored. Supporting the possible importance of global regulation of gene expression in neurodevelopmental disorders, genetic studies found that mutations in genes encoding for global regulators of gene expression were linked to neurodevelopmental disorders including autism [5], [6]. Pharmacological studies also suggested that targeting global levels of gene expression regulation could impact neurodevelopment. For instance, valproate, a histone deacetylase inhibitor (HDACi), is a commonly used medication in the treatment of seizures, mental health disorders, and cancer that impacts global levels of gene expression regulation through chromatin based mechanisms. When given during gestation, valproate can adversely impact neurodevelopment in rodents and cause autism in humans [10], [11], [12], [13]. Thus, both genetic and pharmacological studies suggest alterations in global levels of gene expression regulation can interfere with normal neurodevelopment. Additional studies of various HDAC inhibitors in rodents have shown that HDAC inhibitors may act by altering levels of synaptic plasticity and in this context HDAC inhibitors have been used to modify learning, memory, and emotional behavior underscoring the potentially pleiotropic effects of targeting global levels of gene expression regulation [14], [15], [16], [17], [18], [19].

Addressing the impact of variability in global levels of gene expression regulation on neurodevelopment in mice, we recently reported that the pattern of the distribution of gene expression levels, as assessed by variance in the distribution, accurately predicted mouse behavior in genetically identical animals. Specifically, we found that increased variance across the total distribution of gene expression levels in the hippocampus predicted increased levels of open-field exploration, a hippocampal dependent behavior. Developmental epigenetic interventions that modified the variance in the gene expression distribution in the hippocampus also modified mouse behavior in tandem [20].

**Figure 1:**
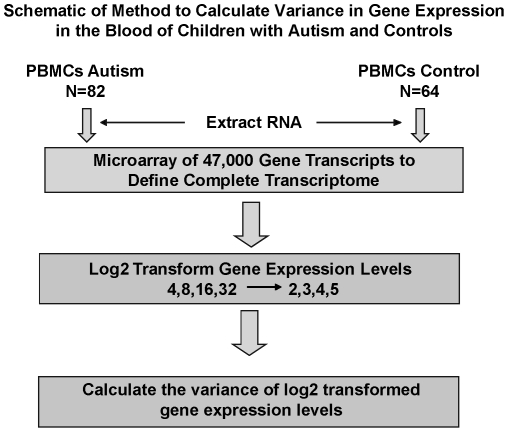
Schematic for determining the large-scale organization of gene expression in the blood of children with autism and controls. The figure displays the steps used to evaluate the large-scale organization of gene expression in the blood of children with autism and controls. Peripheral blood lymphocytes were purified from the blood of children with autism (n = 82) or control subjects (n = 64). Total RNA was extracted for microarray experiments with Affymetrix Human U133 Plus 2.0 3′ Expression Arrays. Array data was pre-processed and summarized using Affymetrix Microarray Analysis Suite 5.0 (MAS 5.0) to obtain 54,675 expression levels for approximately 47,000 unique transcripts including approximately 38,500 human genes. Expression levels were log2 transformed and the variance of log2 transformed expression levels was determined.

In the current study, we used our previously established strategy of studying the overall pattern of the gene expression distribution to test the hypothesis that autism would be associated with alterations in global levels of gene expression regulation. To do this, we compared the pattern of the gene expression distributions, as assessed by variance across the total gene expression distribution, in the blood of children with autism and controls (schematic in figure 1). Further, since multiple studies associated increased paternal age with increased rates of autism and other neurodevelopmental disorders [21], [22], [23], [24], [25], [26], [27], [28], we performed a secondary analysis to address the hypothesis that paternal age would be associated with changes in the pattern of the gene expression distribution. Finally, we used traditional approaches to gene expression analysis to examine for biological pathways that might be causally related to changes global levels of gene expression regulation.

## Materials and Methods

### Subjects

The Western Institutional Review Board approved all aspects of the current protocol related to human subjects including written informed consent and found that the research was conducted according to the principles expressed in the Declaration of Helsinki. All subjects were male. Probands and controls were all recruited from the Phoenix area via database mailing for probands and via flyers to scout, church, and sports groups for controls. Blood draws for all subjects were done between the spring and summer of 2004. Children with autism had a diagnosis of autism by a medical professional (developmental pediatrician, psychologist, or child psychiatrist) according to the DSM-IV criteria, and confirmation of the diagnosis by ADOS [29] and ADI-R criteria [30]. Exclusion criteria included a significant prenatal history (prematurity <35 weeks, intraventricular hemorrhage, severe asphyxia, or cerebral palsy), serious CNS abnormality, or known genetic or metabolic disorder. In an attempt to obtain a homogenous population of children with autism, non-classic forms of autism were excluded, including autism with regression and Asperger's syndrome, a higher functioning form of autism where individuals have language skills within the normal range. In addition, each subject had a normal high-resolution chromosome analysis, and a negative Fragile X DNA test. 82 children with autism and 64 control children were included in the study. IQ scores were not done for this study, but all individuals did have a demonstrated impairment in language as part of the diagnostic criteria. For our secondary analysis, paternal ages were available for 78 children with autism and 57 controls. The group of children with autism was significantly younger than the control group (autism: mean - 5.5 years SD - 2.1; control: mean - 7.9 SD - 2.2, p<.0001). Paternal age was similar between groups. The study population was primarily Caucasian and there were no group level differences in ethnicity.

### Expression profiling

Expression profiling was performed at Translational Genomics (TGen), a member of the NIMH Neuroscience Microarray Consortium. Total RNA was extracted from peripheral blood lymphocytes (PBL) within 30 minutes of the blood draw using the Qiagen Qiaquick kit (Germantown, MD). Isolated total RNA was double round amplified, cleaned, and biotin-labeled using Affymetrix's GeneChip Two-Cycle Target Labeling kit (Santa Clara, CA) with a T7 promoter and Ambion's MEGAscript T7 High Yield Transcription kit (Austin, TX) as per manufacturer's protocol. Amplified and labeled cRNA was quantified on a spectrophotometer and run on a 1% TAE gel to check for an evenly distributed range of transcript sizes. Twenty micrograms of cRNA was fragmented to approximately 35–200 bp by alkaline treatment (200 mM Tris-acetate, pH 8.2, 500 mM KOAc, 150 mM MgOAc) and run on a 1% TAE gell to verify fragmentation. Separate hybridization cocktails were made using 15 micrograms of fragmented cRNA from each sample as per Affymetrix's protocol.

Two hundred microliters (containing 10 micrograms of fragmented cRNA) of each cocktail was separately hybridized to an Affymetrix Human Genome U133 Plus 2.0 Array for 16 h at 45 degree Celsius in the Hybridization Oven 640. The Affymetrix Human Genome Arrays measure the expression of over 47,000 transcripts and variants, including 38,500 characterized human genes. Arrays were washed on Affymetrix's GeneChip Fluidics Station 450 using a primary streptavidin phycoerythrin (SAPE) stain, subsequent biotinylated antibody stain, and secondary SAPE stain. Arrays were scanned on Affymetrix's GeneChip Scanner 3000 7G with AutoLoader. Scanned images obtained by the Affymetrix GeneChip Operating Software (GCOS) v1.2 were used to extract raw signal intensity values per probe set on the array. A scaling factor of 150 was used to normalize array signal intensity in MAS 5.0.

Arrays were scanned over 1 day on 2 different machines. Arrays scanned on the same machine and in the same day were considered to be from the same scan batch. Rescanning of a limited number of samples indicated that there were no significant differences between machines, nonetheless, for all comparisons groups were balanced for the scan batch. Gene expression levels were not adjusted for possible batch effects as algorithms that attempt to adjust for batch effects also alter the gene expression distribution. When samples could not be prepped simultaneously they were balanced for group membership (autism vs. control). To statistically control for possible confounds related to scan batches in our analysis of gene expression variance, batch number was entered into an analysis of covariance. For traditional analysis of gene expression, experimental groups were balanced with respect to batch membership.

### Microarray data analysis

Affymetrix .cel files were imported into Affymetrix Expression Console version 1.1. Data was pre-processed and summarized by Microarray Analysis Suite (MAS) 5.0 and Robust Multiarray Analysis (RMA). For the analysis of gene expression distributions, MAS 5.0 was used because the algorithm does not alter the gene expression distribution, whereas, RMA utilizes quantile normalization of probes prior to summarization and, therefore, has the potential to remove group level differences in gene expression distributions. Because of the numerous advantages in its handling of noise in the measurement of gene expression levels and background subtraction, RMA was used for traditional gene expression analyses looking for specific gene expression differences between groups. Because group level differences in the distribution of gene expression levels between groups were found, gene expression levels were also quantile normalized after the summarization step for traditional gene expression analyses. Quantile normalization adjusts all data sets such that they have identical distribution patterns. Probe sets were then filtered for those that were called present in at least 50 out of the 146 subjects (n = 25,146 probe sets). A p-value of .05 was used as a threshold for significance. A fold-change of 1.1 was used as a cut off for magnitude of change. All microarrays met manufacturers recommended quality control criteria. Present calls ranged from 37.4% to 49%, mean 43.7%, SD 2.7%. Actin 3′to5′ ratios ranged from .726 to 5.15, mean1.37, SD 0.5. There were no significant group level differences in quality control measures.

### Variance across the distribution of gene expression levels

Variance across the distribution of gene expression levels was the primary measure used in the present study. The measure of overall variance compresses data from the expression levels of 54,675 probe sets covering approximately 47,000 unique transcripts including 38,500 human genes on the Affymetrix Human HG-U133 Array into a single variable. We previously found that overall variance in gene expression levels in the hippocampus of mice accurately predicted hippocampal-dependent behavior[20]. To calculate overall variance, gene expression levels for all 54,675 probe sets on the Affymetrix Human HG-U133 Array were log2 transformed. Variance in log2 transformed expression levels was calculated across all 54,675 probe sets in the total distribution as the average of the squares of the deviations from the mean.

### Pathway Analysis

DAVID (david.abcc.ncifcrf.gov) Functional Annotation Bioinformatics Microarray Analysis[31], [32] was used to assess gene lists for enriched biological themes. A user supplied background list was uploaded which consisted of the list of all probe sets that were called present by Affy MAS 5.0 in at least 50 of 146 subjects (n = 25,146). Lists of significant probe sets met the following criteria: present in at least 50 subjects, p<.05, and absolute fold change of at least 1.1-fold.

### Statistical Cross Validation

We found significant differences in variance in gene expression between children with autism and controls. When the paternal age of the control father was considered it was found that variance in gene expression of children with autism was only significantly different from children of younger fathers. Additionally, we found that variance in gene expression was significantly different between children of older fathers and children of younger fathers. To validate these findings we subdivided our data sets into 6 subgroups to allow for within study “replications”. In all cases subgroups of the same type (e.g. autism, younger father, older father) were equally balanced for paternal age and scan batch. The validation sets were as follows: Autism1 (n = 41), Autism2 (n = 41), Control young father1 (n = 14), Control young father2 (n = 14), Control old father1 (n = 14), Control old father2 (n = 14), Control 1 (n = 28 - combination of control young father1 and control old father1), and Control 2 (n = 28 - combination of control young father2 and control old father2)(figure S1 and table S1).

### Transcription inhibitor dataset

In order to assess for changes in gene expression variance in response to globally targeting transcription, we downloaded a publically available dataset in which a new class of transcription inhibitors, sappyrins, were compared to actinomycin D. The microarray study analyzed gene expression in A549 human lung cancer cells that were treated with transcriptional inhibitors[33]: [actinomycin D (5 ug/mL), sapphyrin (low – 1.5 uM), sapphyrin (high – 2.5 uM), or mannitol (5%) as a control]. Cel files were downloaded from the Gene Expression Omnibus (http://www.ncbi.nlm.nih.gov/geo) public database. The microarrays used in the transcription inhibitor study were the same used in the current study, Affymetrix Human Genome U133 Plus 2.0 Array. Microarray data was pre-processed and summarized as described above using Expression Console and the MAS 5.0 algorithm.

### Statistical Analyses

JMP8 and Statview software (SAS, Cary, NC) were used for statistical analyses. The statistical analyses used in the study are listed below.

### Tests of normality

To determine if parametric tests were appropriate, the Shapiro-Wilk Test was performed for the primary measures: variance in gene expression and paternal age. When assessed across the entire study or across all control subjects, variance in gene expression was not normally distributed, but was normally distributed within experimental groups: 1) autism, 2) children of older fathers, and 3) children of younger fathers. Paternal age was normally distributed across the entire study population and within relevant experimental groups: 1) autism and 2) controls. Based on these analyses of normality, parametric tests were used when appropriate.

### Pearson's correlation coefficient (r)

Pearson's correlation coefficient (r) between two variables is defined as the covariance of the two variables divided by the product of their standard deviations. The square of the Pearson's correlation coefficient (R^2^) estimates the proportion of variance in a dependent variable (e.g. overall variance) that is accounted for by an independent variable (e.g. paternal age). Fisher's transformation was used to calculate a p-value from the Pearson's r. For our analyses we considered a p-value of .05 to be statistically significant.

### Spearman's rank correlation (rho)

Spearman's rank correlation assesses how well the relationship between two variables can be described using a monotonic function. It is used to assess the relationship between 2 variables when data is not normally distributed.

### Unpaired Student's T-tests

Compares two groups of normally distributed data to test whether the means of the distributions are different. The p-value represents the probability that the distributions are actually different. For our analyses we considered a p-value of .05 to be statistically significant.

### Analysis of covariance

Analysis of covariance was used to examine the potential confounding effects of subject age and scan batch on a significant association of autism with decreased overall variance. Variance was the dependent variable, diagnosis (−1 =  autism, +1 =  control), subject age, and scan batch (−1 =  batch 1, +1 =  batch 2). All possible interaction terms were also included in the model. For our analyses we considered a p-value of .05 to be statistically significant.

### Chi square

An internet based 2×2 chi square contingency table (http://www.graphpad.com/quickcalcs/contingency1.cfm) was used to assess for a significant overlap between gene lists. A chi-square with Yates correction was used to calculate chi squared and a two-tailed p-value.

## Results

### Decreased overall variance in log-transformed measures of gene expression predicts the diagnosis of autism (figures 1 and 2)

**Figure 2:**
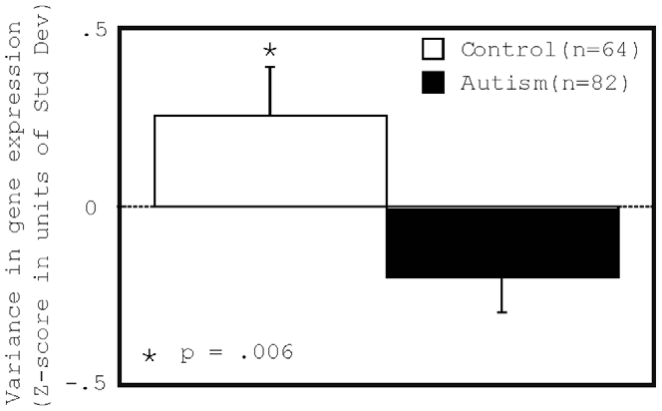
Overall variance in gene expression in peripheral blood lymphocytes (PBL) was decreased in children with autism. We used microarrays to measure the expression levels of greater than 47,000 transcripts including 38,500 well-characterized human genes using the Affymetrix Human U133 Plus 2.0 microarray on RNA from peripheral blood lymphocytes from each of 82 children with autism and 64 control subjects. Microarrays showed no group level differences in quality control measures. Microarray expression levels were log-transformed and the overall variance was calculated across the total distribution of expression levels on each microarray. Variance in gene expression was significantly decreased in the blood of children with autism (p = .006). Error bars represent standard error.

We used microarrays to measure the expression levels of greater than 47,000 unique transcripts including 38,500 well-characterized human genes using the Affymetrix Human U133 Plus 2.0 microarray with purified RNA from peripheral blood lymphocytes from each of 82 children with sporadic cases of autism and 64 control subjects (figure 1). In contrast to comparing the expression levels of individual genes, we compared the pattern of the overall distribution of gene expression levels between children with autism and controls. Measurement of the variance in the distribution of gene expression levels assessed for differences at the global level of gene expression regulation. To obtain a normal-like distribution, gene expression levels were log2-transformed. The overall variance of the total distribution was measured for each microarray (schematic in figure 1). The distribution of gene expression levels on microarrays from individuals with autism had a decreased variance when compared to microarrays from controls (figure 2, p = .006, parameter estimate  = −.45 Std Dev lower in autism).

Because the group of children with autism was significantly younger than the control group (autism: mean - 5.5 years SD - 2.1; control: mean - 7.9 SD - 2.2, p<.0001), we tested whether subject age had an effect on the relationship between diagnosis and overall variance. An analysis of covariance (ANCOVA) demonstrated that even when controlling for subject age, variance in gene expression continued to be significantly decreased by the same amount in the blood of children with autism compared to controls (p = .018, parameter estimate  = −.45 Std Dev lower in autism). When scan batch was included with subject age in the ANCOVA the results also remained significant (p = .03, parameter estimate  = −.42 Std Dev lower in autism). Importantly, parameter estimates for the relationship of diagnosis to variance were virtually unchanged in the ANCOVAs indicating that increasing p-values were related to increases in the degrees of freedom in the analysis, and not to a decreased association.

### Increased paternal age is associated with decreased overall variance in gene expression levels (figure 3)

**Figure 3:**
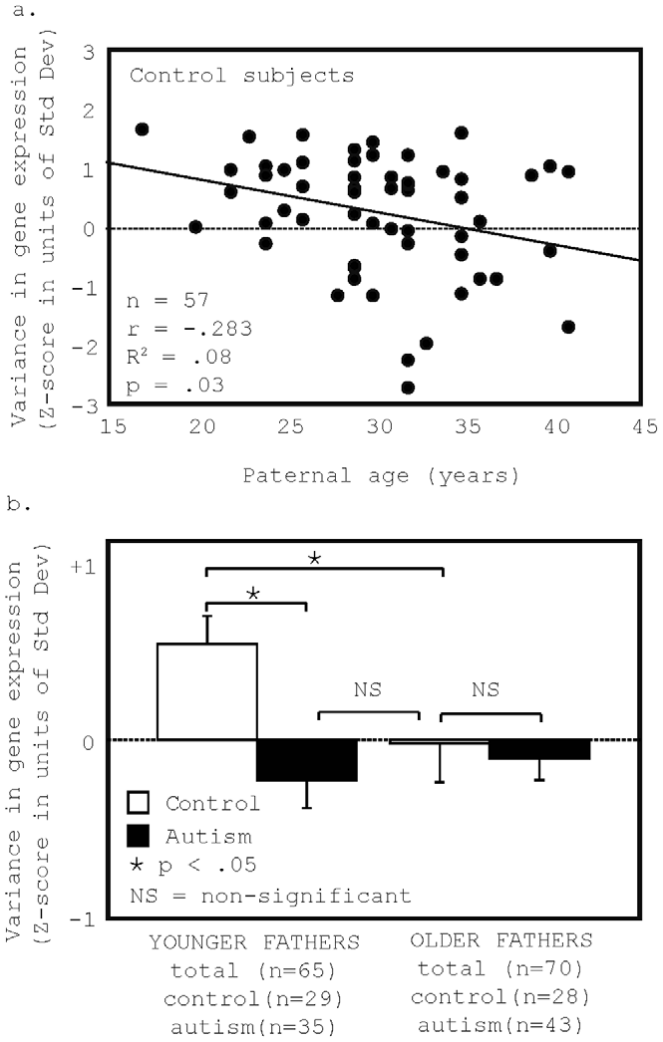
Increased paternal age at birth is negatively associated with overall variance in gene expression in peripheral blood lymphocytes (PBL) of normal children. Paternal age at birth was found in multiple studies to be a risk factor for autism [26], [28], [34], [35]. Previous work indicated that factors or interventions that modified mouse hippocampal-dependent behavior also modified the overall variance in gene expression in the predicted direction. In controls (figure 3a) but not in children with autism (not shown), overall variance in log-transformed measures of gene expression was significantly and negatively associated with paternal age at birth (r = -.283, R^2^ = .08, p = .03, number of subjects  = 57). For the evaluation of paternal age effects, paternal ages were available for 78 children with autism and 57 control children. To directly compare overall microarray variance in children with autism to children of older fathers, we divided subjects by the median paternal age at birth in our study (31 years) and created 2 groups: 1) children from younger fathers (less than 31 years) (65 subjects: 30 controls and 35 children with autism); and 2) children from older fathers (31 years or older) (70 subjects: 27 controls and 43 children with autism). We compared mean levels of overall variance between children with autism and controls of older and younger fathers. As predicted, we found that overall variance was the same in children of older fathers and children with autism with fathers of any age (figure 3b). We found that the association of autism with decreased overall variance was only found in children of younger fathers and represented a difference of .78 standard deviations within our sample (p = .0007) (figure 3b). Error bars represent standard error.

Previous work in mice indicated that factors or interventions that modified mouse hippocampal-dependent behavior also modified overall variance in the predicted direction [20]. We hypothesized, therefore, that factors that modified risk for autism in the general population might alter the variance in gene expression in a manner that resembled the variance in gene expression in children with autism. Paternal age was found in multiple studies to be a risk factor for autism and other neurodevelopmental disorders such as schizophrenia and mental retardation [26], [28], [34], [35]. We found that in controls, but not in children with autism, overall variance in gene expression was significantly and negatively associated with paternal age (Pearson r = −.283, R^2^ = .08, p = .03, parameter estimate  = −.054 Std Dev/year of paternal age) (figure 3a). Using an analysis of covariance (ANCOVA) results remained significant after controlling for subject age (p = .046, parameter estimate  = −.052 Std Dev/year of paternal age) and scan batch (p = .03, parameter estimate  = −.055 Std Dev/year of paternal age). When both potential confounds were entered into the model the parameter estimate remained unchanged, but the p-value was slightly above .05 (p = .056, parameter estimate  = −.052 Std Dev/year of paternal age). Thus, paternal age-related changes in gene expression variance in control subjects were similar in direction to autism-related changes in variance. We also assessed the relationship between paternal age and variance in gene expression using Spearman rank correlation (Spearman rho  = −.274, rho corrected for ties  = −.281, p = .04, p corrected for ties  = .036). The use of a non-parametric test addressed any potential concerns about the effects of possible outliers (though no values were greater than 3 standard deviations from the mean) and of problems arising from the use of non-normal data distributions with parametric tests. The measurement of variance in the distribution of gene expression levels was not normally distributed within the group of control children.

Because increased paternal age and autism were both negatively related to overall variance in gene expression, it was predicted that the overall variance in gene expression for children of older fathers would be similar to that in children with autism. To confirm this hypothesis, we performed a median split of subjects by paternal age (median  = 31 years) and assessed for differences in overall variance in gene expression between children with autism and controls in groups of children from older and younger fathers. As predicted, we found that the overall variance was the same in the blood of children from older fathers as in the blood of children with autism from fathers of any age (figure 3b). We found that the association of autism with decreased overall variance was only found in children of younger fathers (p = .0007) (figure 3b). As expected from the negative correlation, in the median split analysis, variance in gene expression in the blood of control children of older fathers was significantly decreased compared to control children of younger fathers (p = .03) (figure 3b).

### Decreased variance in gene expression is related to the down-regulation of genes involved in the regulation of transcription (figure 4 and tables S2–S4)

**Figure 4:**
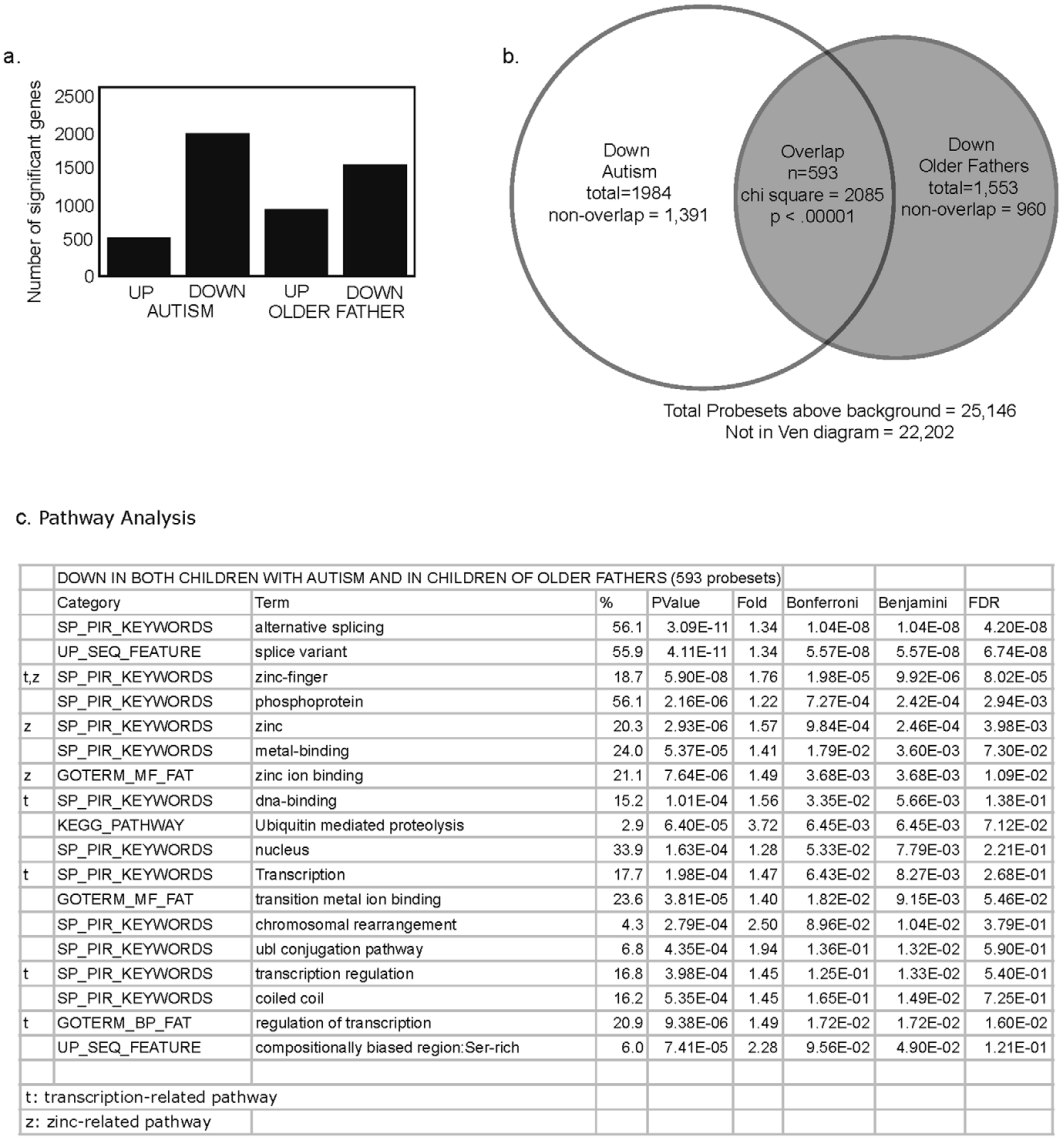
Down-regulated genes are enriched for biological pathways related to transcription and zinc. Traditional gene expression analyses were performed. Microarrays were processed and summarized using RMA. Post-summarization gene expression levels were quantile normalization because of the known group level differences at the level of the distribution. Probe sets were filtered for those that were called present in at least 50 of 146 samples. Figure 4a shows that there were more down-regulated than up-regulated genes in the blood of children with autism and children of older fathers when compared to children of younger fathers. 4b shows a very significant overlap (chi-squared  = 2,085, p<.00001) of down-regulated genes in children with autism and children of older fathers. Figure 4c demonstrates that overlapping down-regulated genes are enriched for biological pathways associated with transcription and zinc.

To evaluate for a possible mechanism underlying decreased variance in gene expression in the blood of children with autism or from older fathers, we analyzed for specific gene expression differences between study groups. Gene expression patterns in the blood of children with autism and control children of older fathers were compared to gene expression patterns in the blood of control children of younger fathers. First, it was noted that in the blood of children with autism and children of older fathers, there were many more significantly down-regulated genes than up-regulated genes (figure 4a). In the comparison of children with autism to children of younger fathers, there 2,093 genes that were significantly down-regulated by at least 1.1-fold and only 641 genes that were up-regulated. In the blood of children of older fathers compared to children of younger fathers there were 1,476 down-regulated and 764 up-regulated genes. To determine if the same genes were up or down-regulated in children with autism and children of older fathers, we compared lists of genes and assessed the degree of overlap. The overlaps between genes that were down or up-regulated in children with autism and children of older fathers were highly significant. There were 593 genes that were down-regulated in both children with autism and children of older fathers (figure 4b) (chi square 2,071, p<.000001) and 145 genes that were both up-regulated in both comparisons (chi square 849, p<.000001).

To determine if these overlapping genes were enriched for particular biological pathways, DAVID was used to perform pathway analysis. As background, we used the 25,146 probe sets that were above our background cut-off requiring that they were called present in at least 50 out of 146 samples. Pathway analysis indicated that multiple pathways associated with transcriptional regulation and supported by multiple independent databases, e.g. GOTERM, BIOCARTA, OMIM disease, etc., were enriched within the shared set of genes that were down-regulated in children with autism and children of older fathers (figure 4c). Importantly, we also found that the same pathways were enriched in the lists of genes that were significantly down-regulated but not shared between children with autism and children of older fathers (Table S2), i.e. there was overlap in biological pathways even when the specific genes were not identical. Significant pathways associated with up-regulated genes (Table S3) were fewer in number and did not appear to offer any mechanistic understanding for global changes in gene expression regulation. Pathway analysis of gene lists obtained using more stringent cutoffs (51% present and a 1.25-fold change) highlighted many of the same pathways related to transcriptional regulation even though the gene lists were much shorter (72 compared to 593 genes in the list of overlapping down-regulated genes) (table S4).

### Cross-validation (figure S1 and table S1)

To evaluate the robustness of our findings, experimental groups were sub-divided to perform statistical cross-validation. For instance, the group of 82 children with autism was divided into 2 groups of 41 children that were balanced for paternal age and microarray scan batch (Autism1 and Autism2), the group of children with older fathers (>30 years old) was divided into 2 groups of 14 children (Old father1 and Old father2), and the group of children of younger fathers (< or  = 30 years old) was divided into 2 groups of 14 children (Young father1 and Young father2). With respect to variance in gene expression (Figure S1), cross-validation of autism vs. children of young fathers remained significant in both analyses, whereas the difference between children of older fathers and children of younger father was no longer significant (Figure S1). Cross-validation using Pearson correlations found that paternal age was significantly correlated within the Conrol1 group (p = .02) but not in the Control2 group. The lack of significance in the cross-validation of paternal age effects appeared to be related to decreased power because of the smaller number of subjects per group in the analysis of age effects, as the means of the subgroups were similar to those of children with autism (figure S1).

Cross-validation of pathway analysis indicated that these findings were highly robust. Significant gene lists from subgroups (Autism1 vs. Young father1, Autism2 vs. Young father2, Old father1 vs. Young father1, and Old father2 vs. Young father2) indicated that in all cases, significantly down-regulated genes in children with autism or children of older fathers were enriched for genes involved in transcription (Table S1). It was also noted in these analyses that, in all cases, sets of down-regulated genes were also enriched for zinc finger C2H2 proteins and for genes involved in zinc pathways. Zinc has a well-documented role in neurodevelopment[36], [37], [38] and also plays any important role in the stabilization of many proteins involved in transcription[38], suggesting that zinc-mediated effects on transcription may be particularly important in contributing to global effects on gene expression.

### Inhibition of transcription leads to a decreased variance in gene expression (figure 5)

**Figure 5:**
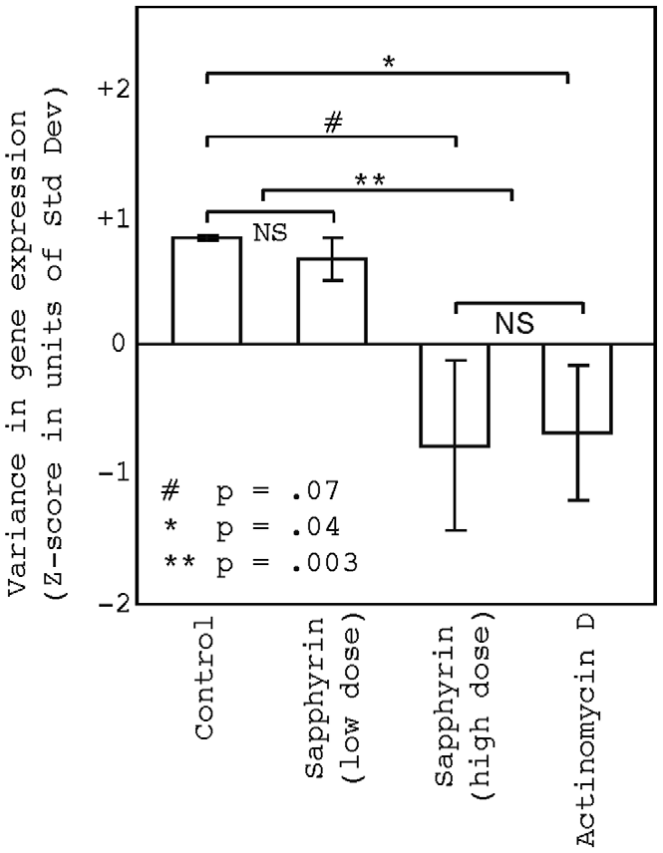
Transcription inhibitors lead to decreased variance in gene expression. Gene expression data from a publically available dataset show that treatment of a lung cancer cell line with actinomycin D and a high dose of sapphyrin results in decreased variance in gene expression compared to treatment with mannitol or a low dose of sapphyrin. Error bars represent standard error.

Pathway analysis showed that regulators of transcription were enriched in the subset of genes that were down-regulated in children with autism and control children of older fathers. This suggested that dysregulation of transcription might be a mechanism underlying the decreased variance in gene expression in these groups of children. To explore this hypothesis, we used a publically available dataset to examine whether inhibition of transcription can lead to decreased variance in gene expression. In the gene expression study, a human lung cancer cell line, A549, was treated with transcription inhibitors for four hours. Treatment with high levels of sapphyrin (n = 3, p = .07), a new class of transcription inhibitor, or actinomycin D (n = 3, p = .04) led to a decrease in variance in gene expression when compared to control treatment with mannitol (figure 5). Low levels of sapphyrin had no effect on variance in gene expression. When study groups were pooled to increase power, actinomycin D (n = 3) + high sapphyrin (n = 3) vs. low sapphyrin (n = 3) + mannitol, (n = 3), the effect of transcription inhibition was a highly significant decrease in variance in gene expression (p = .003) (figure 5).

## Discussion

Studying the variance in the distribution of gene expression represents a new approach to gene expression analysis. Because changes in a limited number of genes could not account for a change in the shape of the entire gene expression distribution, changes in variance suggest alterations at global levels of gene expression regulation. This “birds-eye view” of gene expression levels contrasts with gene-centered approaches that seek to identify specific genes or pathways that differ between groups. While gene specific approaches are useful they may miss changes occurring at the global level of gene expression regulation. The two approaches, therefore, represent distinct yet potentially complimentary approaches to understanding the full magnitude of effects on gene expression regulation. Here we used our novel approach of studying the gene expression distribution to associate decreased variance in gene expression levels with the diagnosis of autism and a risk factor for autism, increased paternal age. We complimented this global approach with gene specific analyses that indicated that changes in global levels of gene expression regulation might be related to large-scale changes in transcriptional regulation. Finally, we reinforced the potential role of transcription regulation by demonstrating with a publically available dataset that inhibition of transcription could, in fact, lead to decreased variance in the distribution of gene expression levels.

Global levels of gene expression regulation may be particularly important in understanding the pathological basis of diseases such as autism where multiple systems are affected. This is true because alterations at the global level of gene expression regulation could be shared across systems even when the tissue specific genes affected by these global changes were different. For instance, in the current study, even though gene expression patterns in the brain and blood are known to be different, it is possible that in the brains of children with autism, variance in gene expression is decreased as it is in the blood. Thus, it is possible that a common mediator, a change at the global level of gene expression regulation, could offer a unifying explanation for multi-systemic effects of disease. By understanding that global levels of gene expression regulation can be disrupted in disease, it becomes clear that targeting global levels of gene expression regulation might also be therapeutic, an idea strongly supported by successful treatments such as HDAC inhibitors in mental health disorders and cancer[39]. Interestingly, HDAC inhibitors are known to affect immune, endocrine, and neurological systems, thus giving added support to the idea that globally targeting gene expression regulation can affect multiple systems [14], [39], [40], [41].

The association of paternal age with overall variance in gene expression in the blood of healthy controls is interesting. In our study, decreased variance in gene expression was a risk factor for the diagnosis of autism. That another well established risk factor for autism, increased paternal age, was associated with a similar decrease in variance raises the interesting possibility that the associated increased risk of autism in children of older fathers could be mediated by changes in global levels of gene expression regulation or by paternally transmitted age-related factors that are linked to changes in the global regulation of gene expression. In other words, an effect on global levels of gene expression regulation transmitted by older fathers might act to increase the likelihood that neurodevelopmental abnormalities will occur and thus increase the rates of autism in children of older fathers. Determining whether this hypothesis is correct would require a large-scale prospective study in which statistical models assessed whether increased rates of autism in children of older fathers were statistically mediated by effects on variance in gene expression.

The association of variance in gene expression with paternal age is also interesting because it suggests that fathers may transmit factors influencing global levels of gene expression regulation in offspring. Paternal age has previously been associated with another genomic phenotype, increased telomere length, indicating that, in principal, global genomic properties can be sensitive to paternal age[42], [43]. Possible mechanisms for the transmission of factors influencing global levels of gene expression regulation are suggested by the association of aging with *de novo* germ line mutations and global changes in DNA methylation [44]. Each of these properties, if paternally transmitted to offspring, could influence global regulation of gene expression. Given the vast number of gene expression changes that occur throughout development, understanding how global effects on gene expression regulation may be transmitted across the germ line to ultimately influence the overall variance in gene expression in differentiated cells could prove crucial to our understanding of how general genomic properties are transmitted to and influence the function of very different cell types.

The parallels of the current study to our work in mice also bear discussion. We found in mice that epigenetic factors acted during early brain development to elicit changes in adult behavior that were accompanied by changes in the variance in gene expression[20]. In a separate study, we found that the same hippocampal-dependent behaviors were paternally transmitted to offspring even though fathers were not present for rearing suggesting an epigenetic germ line transmission of factors that influenced brain development, behavior, and variance in gene expression [45]. In autism, paternally transmitted epigenetic factors related to age have also been implicated [23], [26] and in the current study paternal age was associated with differences in the variance in gene expression in the general population. Though much further study is need, both lines of work suggest the presence of epigenetic paternally transmitted factors in both mice and humans that can influence brain development and global levels of gene expression regulation.

Alterations in the overall variance in gene expression in peripheral blood lymphocytes are also of interest. Children with autism have immunological abnormalities and it has been previously reported that gene expression differences were found in immune cells of children with autism [8], [9], [46]. More surprising was the finding in this study that increased paternal age was associated with the same global gene expression phenotype and specific alternations in transcription-related biological pathways in healthy controls as was associated with the diagnosis of autism. As discussed, a relationship to paternal age suggests that factors influencing global levels of gene expression regulation may be transmitted across the germ line. Additionally, findings in the blood suggest the possibility for effects of paternal age on immune function. An association of increased paternal age with immune function is suggested by a common link between increased paternal age and mental disorders such as schizophrenia, bipolar disorder, and autism, and the association of these disorders with a common set of immune abnormalities that includes increases in pro-inflammatory cytokines, IL-1beta and IL-6 [47], [48]. A more generalized effect of paternal age on immune function seems plausible. Increased paternal age has been associated with impairments in neurocognitive ability during infancy and childhood in the general population [34] and with an increase in “externalizing” behaviors in the general population [49]. Given the links between paternal age, neurodevelopomental disorders, and immune function, it seems that paternal age might also have a more generalized effect on immune function as it was found to have on neurodevelopment.

Finally, traditional approaches to gene expression analysis suggested a possible mechanism for the changes in gene expression variance. We found enrichment for genes involved in transcriptional regulation in genes that were down-regulated with respect to paternal age and autism. Analysis of the effects of transcription inhibition on gene variance was supportive of the hypothesis that down-regulation of transcriptional regulators may account for the decreased variance in gene expression in the current study. Also of interest we found enrichment for genes regulated by zinc, an important component of many transcription factors. Since it has been demonstrated that neuronal plasticity is often transcription dependent [50], [51], it seems plausible that an alteration in the general regulation of transcription could adversely impact neurodevelopment. Though further study is needed, the suggestion by pathway analysis of transcription dysregulation in autism is consistent with genetic findings that mutations in general transcription factors are associated with neurodevelopmental disorders including autism [5]. The possible importance of zinc is also of interest given the recent report of a decreased zinc/copper ratio in children with autism spectrum disorders [52].

Multiple lines of evidence including genetic linkage [5], [6], pharmacological studies [10], [11], [12], [53], and the associations observed in the current study suggest that variations in global levels of gene expression regulation may contribute to the risk for neurodevelopmental disorders including autism. An association of variance in gene expression with paternal age in healthy controls suggests that factors influencing global levels of gene expression regulation may be transmitted across the germ line. Findings in immune cells suggest that global levels of gene expression regulation may impact systems other than the brain. Finally, pathway analysis suggests that altered regulation of transcription may underlie decreased variance and may increase risk for autism. A more thorough understanding of the mechanisms and the biological impact of the reported associations with overall variance in gene expression represent important areas for future research.

## Supporting Information

Figure S1 **Cross-validation.** Figure describes the work flow used for cross-validation. Briefly, experimental groups were divided in half to create 6 subgroups that were balance for scan batch (autism1, autism2, children of older fathers1, children of older fathers2, children of younger fathers1, children of younger fathers2). a) Shows cross-validation of changes in gene expression variance. The means of autism1, autism2, older father1, and older father 2 are lower than the means of younger father 1 and 2, but only the autism subgroups are significantly different. b) Demonstrates a significant overlap between down-regulated genes in autism1 vs. young father1 and autism2 vs. young father2 (chi-squared  = 866.6, p<.00001). c) Demonstrates a significant overlap in down-regulated genes in old father1 vs. young father1 and old father2 vs. young father2 (chi-squared  = 10.4, p = .0013).
(TIFF)

Table S1 **Cross-validation of pathway analysis of down-regulated genes.** Pathway analysis was done on significant gene lists (p<.05, absolute fold change >1.1) from cross-validation datasets (autism1 vs. younger father1, autism2 vs. young father2, old father1 vs. young father1, old father2 vs. young father 2). The table illustrates that cross-validation datasets highlighted the same enrichment of biological pathways related to transcription and zinc.
(PDF)

Table S2 **Non-overlapping down-regulated genes.** Pathway analysis was done on significant gene lists (p<.05, absolute fold change >1.1) of genes that were down-regulated in autism or in children of older fathers and not overlapping with the other group.
(PDF)

Table S3 **Up-regulated Pathways.** Pathway analysis was done on significant gene lists (p<.05, absolute fold change >1.1) of genes that were up-regulated in autism or in children of older fathers.
(PDF)

Table S4 **Pathway analysis with increased stringency for gene list selection.** Pathway analysis was done on significant gene lists (p<.05, absolute fold change >1.25, and present calls >51%) of genes that were down-regulated in autism and in children of older fathers. Table shows that the same pathways were enriched as with lower stringency gene list selection.
(PDF)

## References

[1] Newschaffer CJ, Croen LA, Daniels J, Giarelli E, Grether JK (2007). The epidemiology of autism spectrum disorders.. Annu Rev Public Health.

[2] Sykes NH, Lamb JA (2007). Autism: the quest for the genes.. Expert Rev Mol Med.

[3] Stephan DA (2008). Unraveling autism.. Am J Hum Genet.

[4] Wang K, Zhang H, Ma D, Bucan M, Glessner JT (2009). Common genetic variants on 5p14.1 associate with autism spectrum disorders.. Nature.

[5] Aso T, Shilatifard A, Conaway JW, Conaway RC (1996). Transcription syndromes and the role of RNA polymerase II general transcription factors in human disease.. J Clin Invest.

[6] Zoghbi HY (2005). MeCP2 dysfunction in humans and mice.. J Child Neurol.

[7] Enstrom AM, Lit L, Onore CE, Gregg JP, Hansen RL (2009). Altered gene expression and function of peripheral blood natural killer cells in children with autism.. Brain Behav Immun.

[8] Gregg JP, Lit L, Baron CA, Hertz-Picciotto I, Walker W (2008). Gene expression changes in children with autism.. Genomics.

[9] Hu VW, Frank BC, Heine S, Lee NH, Quackenbush J (2006). Gene expression profiling of lymphoblastoid cell lines from monozygotic twins discordant in severity of autism reveals differential regulation of neurologically relevant genes.. BMC Genomics.

[10] Ornoy A (2009). Valproic acid in pregnancy: how much are we endangering the embryo and fetus?. Reprod Toxicol.

[11] Schneider T, Przewlocki R (2005). Behavioral alterations in rats prenatally exposed to valproic acid: animal model of autism.. Neuropsychopharmacology.

[12] Wagner GC, Reuhl KR, Cheh M, McRae P, Halladay AK (2006). A new neurobehavioral model of autism in mice: pre- and postnatal exposure to sodium valproate.. J Autism Dev Disord.

[13] Williams G, King J, Cunningham M, Stephan M, Kerr B (2001). Fetal valproate syndrome and autism: additional evidence of an association.. Dev Med Child Neurol.

[14] Lattal KM, Barrett RM, Wood MA (2007). Systemic or intrahippocampal delivery of histone deacetylase inhibitors facilitates fear extinction.. Behav Neurosci.

[15] Tsankova NM, Berton O, Renthal W, Kumar A, Neve RL (2006). Sustained hippocampal chromatin regulation in a mouse model of depression and antidepressant action.. Nat Neurosci.

[16] Weaver IC, Champagne FA, Brown SE, Dymov S, Sharma S (2005). Reversal of maternal programming of stress responses in adult offspring through methyl supplementation: altering epigenetic marking later in life.. J Neurosci.

[17] Levenson JM, Sweatt JD (2006). Epigenetic mechanisms: a common theme in vertebrate and invertebrate memory formation.. Cell Mol Life Sci.

[18] Moretti P, Levenson JM, Battaglia F, Atkinson R, Teague R (2006). Learning and memory and synaptic plasticity are impaired in a mouse model of Rett syndrome.. J Neurosci.

[19] Fischer A, Sananbenesi F, Wang X, Dobbin M, Tsai LH (2007). Recovery of learning and memory is associated with chromatin remodelling.. Nature.

[20] Alter MD, Rubin DB, Ramsey K, Halpern R, Stephan DA (2008). Variation in the large-scale organization of gene expression levels in the hippocampus relates to stable epigenetic variability in behavior.. PLoS ONE.

[21] Tsuchiya KJ, Matsumoto K, Miyachi T, Tsujii M, Nakamura K (2008). Paternal age at birth and high-functioning autistic-spectrum disorder in offspring.. Br J Psychiatry.

[22] Puleo CM, Reichenberg A, Smith CJ, Kryzak LA, Silverman JM (2008). Do autism-related personality traits explain higher paternal age in autism?. Mol Psychiatry.

[23] Cantor RM, Yoon JL, Furr J, Lajonchere CM (2007). Paternal age and autism are associated in a family-based sample.. Mol Psychiatry.

[24] Croen LA, Najjar DV, Fireman B, Grether JK (2007). Maternal and paternal age and risk of autism spectrum disorders.. Arch Pediatr Adolesc Med.

[25] Miller MC (2006). Older father, autistic child.. Harv Ment Health Lett.

[26] Reichenberg A, Gross R, Weiser M, Bresnahan M, Silverman J (2006). Advancing paternal age and autism.. Arch Gen Psychiatry.

[27] Malaspina D, Corcoran C, Fahim C, Berman A, Harkavy-Friedman J (2002). Paternal age and sporadic schizophrenia: evidence for de novo mutations.. Am J Med Genet.

[28] Malaspina D, Reichenberg A, Weiser M, Fennig S, Davidson M (2005). Paternal age and intelligence: implications for age-related genomic changes in male germ cells.. Psychiatr Genet.

[29] Lord C, Rutter M, Goode S, Heemsbergen J, Jordan H (1989). Autism diagnostic observation schedule: a standardized observation of communicative and social behavior.. J Autism Dev Disord.

[30] Lord C, Rutter M, Le Couteur A (1994). Autism Diagnostic Interview-Revised: a revised version of a diagnostic interview for caregivers of individuals with possible pervasive developmental disorders.. J Autism Dev Disord.

[31] Huang da W, Sherman BT, Lempicki RA (2009). Systematic and integrative analysis of large gene lists using DAVID bioinformatics resources.. Nat Protoc.

[32] Dennis G, Sherman BT, Hosack DA, Yang J, Gao W (2003). DAVID: Database for Annotation, Visualization, and Integrated Discovery.. Genome Biol.

[33] Wang Z, Lecane PS, Thiemann P, Fan Q, Cortez C (2007). Synthesis and biologic properties of hydrophilic sapphyrins, a new class of tumor-selective inhibitors of gene expression.. Mol Cancer.

[34] Saha S, Barnett AG, Foldi C, Burne TH, Eyles DW (2009). Advanced paternal age is associated with impaired neurocognitive outcomes during infancy and childhood.. PLoS Med.

[35] Malaspina D, Harlap S, Fennig S, Heiman D, Nahon D (2001). Advancing paternal age and the risk of schizophrenia.. Arch Gen Psychiatry.

[36] Mackenzie GG, Zago MP, Aimo L, Oteiza PI (2007). Zinc deficiency in neuronal biology.. IUBMB Life.

[37] Adamo AM, Oteiza PI (2010). Zinc deficiency and neurodevelopment: the case of neurons.. Biofactors.

[38] Aimo L, Mackenzie GG, Keenan AH, Oteiza PI (2010). Gestational zinc deficiency affects the regulation of transcription factors AP-1, NF-kappaB and NFAT in fetal brain.. J Nutr Biochem.

[39] Wiech NL, Fisher JF, Helquist P, Wiest O (2009). Inhibition of histone deacetylases: a pharmacological approach to the treatment of non-cancer disorders.. Curr Top Med Chem.

[40] Tao R, de Zoeten EF, Ozkaynak E, Chen C, Wang L (2007). Deacetylase inhibition promotes the generation and function of regulatory T cells.. Nat Med.

[41] Vecsey CG, Hawk JD, Lattal KM, Stein JM, Fabian SA (2007). Histone deacetylase inhibitors enhance memory and synaptic plasticity via CREB:CBP-dependent transcriptional activation.. J Neurosci.

[42] Unryn BM, Cook LS, Riabowol KT (2005). Paternal age is positively linked to telomere length of children.. Aging Cell.

[43] Kimura M, Cherkas LF, Kato BS, Demissie S, Hjelmborg JB (2008). Offspring's leukocyte telomere length, paternal age, and telomere elongation in sperm.. PLoS Genet.

[44] Malaspina D (2001). Paternal factors and schizophrenia risk: de novo mutations and imprinting.. Schizophr Bull.

[45] Alter MD, Gilani AI, Champagne FA, Curley JP, Turner JB (2009). Paternal Transmission of Complex Phenotypes in Inbred Mice.. Biol Psychiatry.

[46] Ashwood P, Van de Water J (2004). A review of autism and the immune response.. Clin Dev Immunol.

[47] Jyonouchi H, Sun S, Le H (2001). Proinflammatory and regulatory cytokine production associated with innate and adaptive immune responses in children with autism spectrum disorders and developmental regression.. J Neuroimmunol.

[48] Drexhage RC, Knijff EM, Padmos RC, Heul-Nieuwenhuijzen L, Beumer W The mononuclear phagocyte system and its cytokine inflammatory networks in schizophrenia and bipolar disorder.. Expert Rev Neurother.

[49] Saha S, Barnett AG, Buka SL, McGrath JJ (2009). Maternal age and paternal age are associated with distinct childhood behavioural outcomes in a general population birth cohort.. Schizophr Res.

[50] Bading H (2000). Transcription-dependent neuronal plasticity the nuclear calcium hypothesis.. Eur J Biochem.

[51] Wiegert JS, Hofmann F, Bading H, Bengtson CP (2009). A transcription-dependent increase in miniature EPSC frequency accompanies late-phase plasticity in cultured hippocampal neurons.. BMC Neurosci.

[52] Faber S, Zinn GM, Kern JC, Kingston HM (2009). The plasma zinc/serum copper ratio as a biomarker in children with autism spectrum disorders.. Biomarkers.

[53] Schneider T, Ziolkowska B, Gieryk A, Tyminska A, Przewlocki R (2007). Prenatal exposure to valproic acid disturbs the enkephalinergic system functioning, basal hedonic tone, and emotional responses in an animal model of autism.. Psychopharmacology (Berl).

