# Supplementary figures and images for: Autism and Increased Paternal Age Related Changes in Global Levels of Gene Expression Regulation
Source: PLoS One. 2011 Feb 17;6(2):e16715. doi: 10.1371/journal.pone.0016715 (PMC3040743; doi:10.1371/journal.pone.0016715)

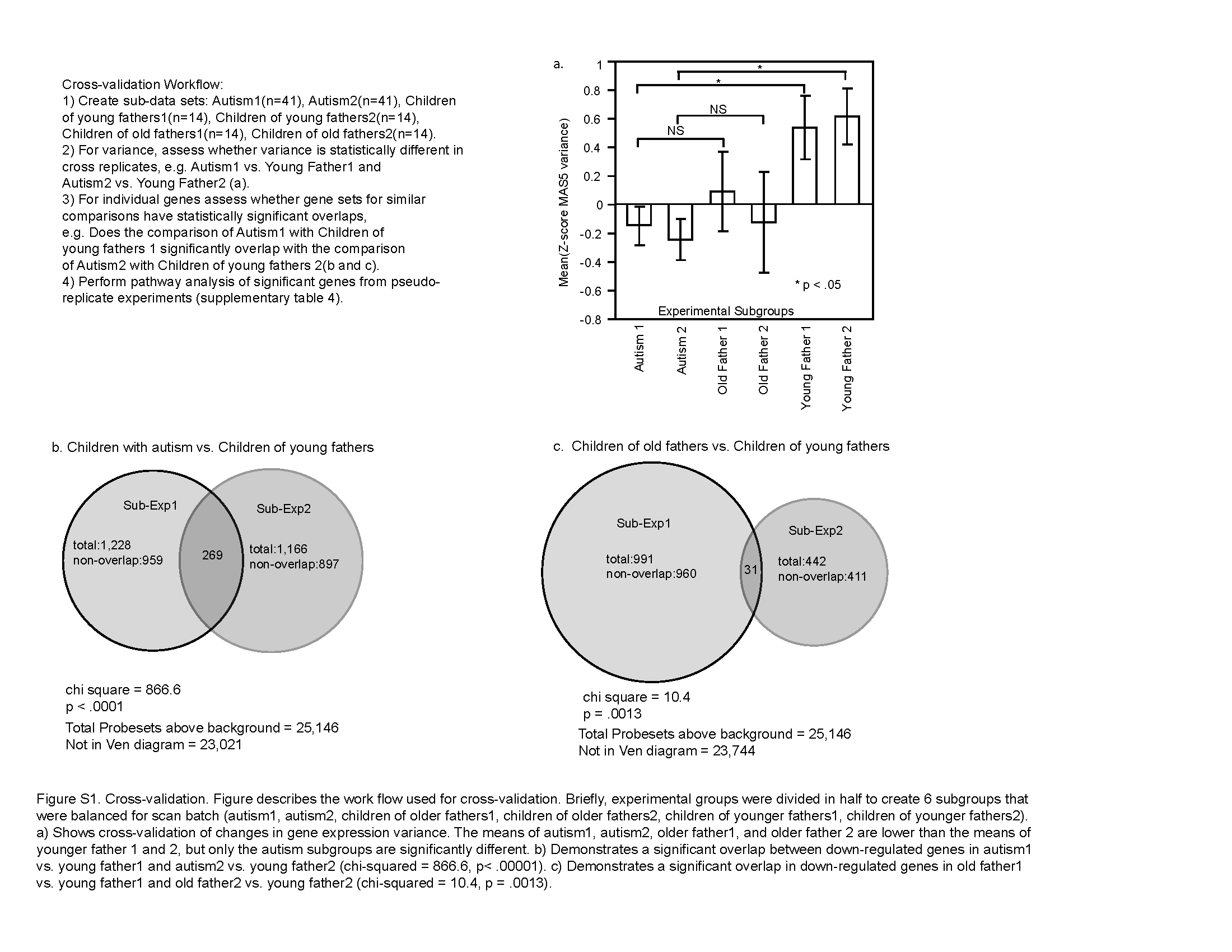

Supplement: Figure S1 — Cross-validation. Figure describes the work flow used for cross-validation. Briefly, experimental groups were divided in half to create 6 subgroups that were balance for scan batch (autism1, autism2, children of older fathers1, children of older fathers2, children of younger fathers1, children of younger fathers2). a) Shows cross-validation of changes in gene expression variance. The means of autism1, autism2, older father1, and older father 2 are lower than the means of younger father 1 and 2, but only the autism subgroups are significantly different. b) Demonstrates a significant overlap between down-regulated genes in autism1 vs. young father1 and autism2 vs. young father2 (chi-squared = 866.6, p<.00001). c) Demonstrates a significant overlap in down-regulated genes in old father1 vs. young father1 and old father2 vs. young father2 (chi-squared = 10.4, p = .0013). (TIFF) [file pone.0016715.s001.tif]
