# Supplementary material for: Autism and Increased Paternal Age Related Changes in Global Levels of Gene Expression Regulation
Source: PLoS One. 2011 Feb 17;6(2):e16715. doi: 10.1371/journal.pone.0016715 (PMC3040743; doi:10.1371/journal.pone.0016715)
Supplement: Table S1 — Cross-validation of pathway analysis of down-regulated genes. Pathway analysis was done on significant gene lists (p<.05, absolute fold change >1.1) from cross-validation datasets (autism1 vs. younger father1, autism2 vs. young father2, old father1 vs. young father1, old father2 vs. young father 2). The table illustrates that cross-validation datasets highlighted the same enrichment of biological pathways related to transcription and zinc. (PDF) [file pone.0016715.s002.pdf]

Table S1: CROSS-VALIDATION OF PATHWAYS ENRICHED IN DOWN-REGULATED GENES

DOWN IN CHILDREN WITH AUTISM 1

	Category	Term	%	PValue	Fold	Bonferroni	Benjamini	FDR
t,z	SP_PIR_KEYWORDS	zinc-finger	15.6	7.37E-07	1.5	3.33E-04	3.33E-04	1.05E-03
t	GOTERM_BP_FAT	transcription	16	1.58E-05	1.4	3.60E-02	3.60E-02	2.76E-02
	UP_SEQ_FEATURE	compositionally biased region:Ser-rich	5	2.35E-05	1.9	4.97E-02	4.97E-02	4.07E-02
	SP_PIR_KEYWORDS	alternative splicing	47.9	2.91E-05	1.2	1.31E-02	6.55E-03	4.13E-02
t	GOTERM_BP_FAT	regulation of transcription	18.1	4.32E-05	1.3	9.54E-02	4.89E-02	7.56E-02
z	GOTERM_MF_FAT	zinc ion binding	17.3	4.96E-05	1.3	3.37E-02	3.37E-02	7.46E-02
	UP_SEQ_FEATURE	splice variant	47.5	6.04E-05	1.1	1.23E-01	6.35E-02	1.05E-01
t,z	INTERPRO	Zinc finger, C2H2-type	7.3	1.50E-04	1.6	1.56E-01	1.56E-01	2.40E-01
z	SP_PIR_KEYWORDS	zinc	16.9	1.54E-04	1.3	6.71E-02	2.29E-02	2.18E-01
t,z	INTERPRO	Zinc finger, C2H2-like	7.3	2.60E-04	1.6	2.55E-01	1.37E-01	4.16E-01
t	GOTERM_BP_FAT	positive regulation of transcription	4.8	2.62E-04	1.8	4.56E-01	1.83E-01	4.58E-01
	GOTERM_BP_FAT	regulation of RNA metabolic process	12.3	2.76E-04	1.4	4.73E-01	1.48E-01	4.82E-01
t	GOTERM_BP_FAT	regulation of transcription, DNA-dependent	12	3.26E-04	1.4	5.31E-01	1.40E-01	5.69E-01
	GOTERM_BP_FAT	positive regulation of RNA metabolic process	4.2	4.41E-04	1.8	6.40E-01	1.57E-01	7.68E-01
t	SP_PIR_KEYWORDS	Transcription	15.6	4.68E-04	1.3	1.91E-01	5.16E-02	6.63E-01
t	GOTERM_BP_FAT	positive regulation of gene expression	4.8	4.84E-04	1.7	6.74E-01	1.48E-01	8.43E-01
t,z	INTERPRO	Zinc finger, C2H2-type/integrase, DNA-binding	5.8	6.46E-04	1.6	5.20E-01	2.17E-01	1.03E+00
	GOTERM_MF_FAT	transition metal ion binding	19.2	6.68E-04	1.2	3.70E-01	2.06E-01	1.00E+00
t	GOTERM_BP_FAT	positive regulation of transcription, DNA-dependent	4	6.82E-04	1.8	7.95E-01	1.79E-01	1.19E+00
	SP_PIR_KEYWORDS	coiled coil	14.5	7.11E-04	1.3	2.75E-01	6.22E-02	1.00E+00
	GOTERM_CC_FAT	Golgi apparatus	7.3	9.64E-04	1.5	3.44E-01	3.44E-01	1.35E+00
t,z	UP_SEQ_FEATURE	zinc finger region:C2H2-type 3	5.8	9.66E-04	1.6	8.78E-01	5.03E-01	1.66E+00

DOWN IN CHILDREN WITH AUTISM 2

	Category	Term	%	PValue	Fold	Bonferroni	Benjamini	FDR
	SP_PIR_KEYWORDS	alternative splicing	53.7	3.72E-16	1.3	1.37E-13	1.37E-13	4.66E-13
	UP_SEQ_FEATURE	splice variant	53.1	4.27E-15	1.3	1.14E-11	1.14E-11	7.68E-12
	SP_PIR_KEYWORDS	phosphoprotein	55.4	3.96E-11	1.2	1.63E-08	8.14E-09	5.54E-08
t,z	SP_PIR_KEYWORDS	zinc-finger	15.9	1.04E-07	1.5	4.26E-05	1.42E-05	1.45E-04
z	SP_PIR_KEYWORDS	zinc	18.2	7.60E-07	1.4	3.12E-04	7.81E-05	1.06E-03
t	SP_PIR_KEYWORDS	Transcription	17	1.64E-06	1.4	6.75E-04	1.35E-04	2.30E-03
t	SP_PIR_KEYWORDS	transcription regulation	16.5	1.85E-06	1.4	7.61E-04	1.27E-04	2.59E-03
t	GOTERM_BP_FAT	transcription	16.8	3.54E-06	1.4	8.95E-03	8.95E-03	6.25E-03
	SP_PIR_KEYWORDS	nucleus	32.6	3.90E-06	1.2	1.60E-03	2.29E-04	5.46E-03
	SP_PIR_KEYWORDS	coiled coil	15.6	9.41E-06	1.4	3.86E-03	4.83E-04	1.32E-02
z	GOTERM_MF_FAT	zinc ion binding	18.9	1.03E-05	1.3	7.51E-03	7.51E-03	1.57E-02
t	GOTERM_BP_FAT	egulation of transcription	19	1.09E-05	1.3	2.72E-02	1.37E-02	1.92E-02
	GOTERM_BP_FAT	regulation of RNA metabolic process	13.4	1.44E-05	1.4	3.60E-02	1.21E-02	2.55E-02
t	SP_PIR_KEYWORDS	dna-binding	13.7	2.18E-05	1.4	8.92E-03	9.95E-04	3.05E-02
	SP_PIR_KEYWORDS	chromosomal rearrangement	3.7	3.63E-05	2.2	1.48E-02	1.49E-03	5.08E-02
t	GOTERM_BP_FAT	regulation of transcription, DNA-dependent	12.9	4.09E-05	1.4	9.86E-02	2.56E-02	7.22E-02
	GOTERM_BP_FAT	M phase	4.2	4.39E-05	2	1.06E-01	2.21E-02	7.76E-02
	UP_SEQ_FEATURE	compositionally biased region:Ser-rich	4.9	5.28E-05	1.9	1.30E-01	6.73E-02	9.36E-02
t	GOTERM_MF_FAT	DNA binding	17.9	5.86E-05	1.3	4.19E-02	2.12E-02	8.89E-02
t	GOTERM_MF_FAT	transcription regulator activity	12	7.35E-05	1.4	5.22E-02	1.77E-02	1.11E-01
	GOTERM_BP_FAT	cell cycle phase	4.9	1.53E-04	1.8	3.22E-01	6.26E-02	2.70E-01
t,z	INTERPRO	Zinc finger, PHD-type	1.9	1.54E-04	2.8	1.63E-01	1.63E-01	2.48E-01
	GOTERM_CC_FAT	nucleoplasm part	5.7	1.76E-04	1.7	7.60E-02	7.60E-02	2.49E-01
t,z	INTERPRO	Zinc finger, PHD-finger	1.8	2.39E-04	2.8	2.42E-01	1.29E-01	3.84E-01
	GOTERM_CC_FAT	nuclear lumen	12.3	2.64E-04	1.4	1.12E-01	5.76E-02	3.73E-01
	GOTERM_BP_FAT	organelle fission	3.2	3.50E-04	2	5.89E-01	1.19E-01	6.16E-01
	SP_PIR_KEYWORDS	mitosis	2.8	3.81E-04	2.2	1.45E-01	1.42E-02	5.33E-01
	GOTERM_BP_FAT	mitosis	3.1	4.00E-04	2	6.38E-01	1.19E-01	7.05E-01
	GOTERM_BP_FAT	nuclear division	3.1	4.00E-04	2	6.38E-01	1.19E-01	7.05E-01
t	GOTERM_MF_FAT	transcription factor activity	7.3	4.50E-04	1.5	2.80E-01	7.87E-02	6.80E-01
	UP_SEQ_FEATURE	compositionally biased region:Lys-rich	2.1	5.49E-04	2.5	7.65E-01	3.83E-01	9.70E-01
	GOTERM_BP_FAT	chromosome organization	5.1	5.50E-04	1.7	7.53E-01	1.44E-01	9.67E-01
	GOTERM_BP_FAT	M phase of mitotic cell cycle	3.1	5.62E-04	2	7.60E-01	1.33E-01	9.88E-01
	SP_PIR_KEYWORDS	metal-binding	20.9	6.10E-04	1.2	2.22E-01	2.07E-02	8.51E-01
	SP_PIR_KEYWORDS	cell division	3.5	7.30E-04	1.9	2.59E-01	2.28E-02	1.02E+00
	SP_PIR_KEYWORDS	rna-binding	5.8	7.93E-04	1.6	2.78E-01	2.30E-02	1.10E+00

t	GOTERM_BP_FAT	negative regulation of transcription	4.4	8.99E-04	1.7	8.98E-01	1.88E-01	1.58E+00
	GOTERM_CC_FAT	nucleoplasm	8	9.62E-04	1.4	3.52E-01	1.34E-01	1.36E+00
	GOTERM_BP_FAT	stem cell differentiation	0.8	9.86E-04	5.5	9.18E-01	1.88E-01	1.73E+00

DOWN IN CHILDREN WITH OLDER FATHERS 1

	Category	Term	%	PValue	Fold	Bonferroni	Benjamini	FDR
	SP_PIR_KEYWORDS	ribonucleoprotein	4.7	2.69E-06	2.2	1.20E-03	1.20E-03	3.81E-03
	GOTERM_BP_FAT	translation	5.1	9.94E-06	2	2.20E-02	2.20E-02	1.73E-02
t,z	SMART	ZnF_C2H2	7.5	1.56E-05	1.7	3.24E-03	3.24E-03	1.97E-02
	GOTERM_BP_FAT	translational elongation	2.4	1.83E-05	3.1	4.03E-02	2.03E-02	3.19E-02
t,z	SMART	KRAB	4.1	1.99E-05	2.2	4.12E-03	2.06E-03	2.50E-02
	GOTERM_CC_FAT	mitochondrial part	7.1	2.06E-05	1.7	8.67E-03	8.67E-03	2.90E-02
	KEGG_PATHWAY	Ribosome	2	2.65E-05	3.4	3.57E-03	3.57E-03	3.10E-02
	GOTERM_CC_FAT	mitochondrial membrane	5.1	3.95E-05	1.9	1.65E-02	8.30E-03	5.55E-02
	GOTERM_CC_FAT	cytosolic large ribosomal subunit	1.3	8.53E-05	4.6	3.54E-02	1.19E-02	1.20E-01
	GOTERM_CC_FAT	mitochondrial envelope	5.2	8.99E-05	1.8	3.72E-02	9.44E-03	1.26E-01
t,z	INTERPRO	Krueppel-associated box	4.1	9.41E-05	2	1.01E-01	1.01E-01	1.51E-01
t,z	INTERPRO	Zinc finger, C2H2-type	7.5	1.06E-04	1.6	1.13E-01	5.84E-02	1.70E-01
t,z	INTERPRO	Zinc finger, C2H2-type/integrase, DNA-binding	6.2	1.30E-04	1.7	1.37E-01	4.81E-02	2.09E-01
	GOTERM_CC_FAT	ribonucleoprotein complex	6.3	1.58E-04	1.7	6.47E-02	1.33E-02	2.23E-01
t,z	INTERPRO	Zinc finger, C2H2-like	7.5	1.82E-04	1.6	1.87E-01	5.04E-02	2.92E-01
	GOTERM_MF_FAT	structural constituent of ribosome	2.8	2.21E-04	2.3	1.34E-01	1.34E-01	3.29E-01
	GOTERM_CC_FAT	ribosome	3.3	2.25E-04	2.1	9.05E-02	1.57E-02	3.15E-01
	SP_PIR_KEYWORDS	nucleus	31.5	2.30E-04	1.2	9.77E-02	5.01E-02	3.26E-01
t,z	UP_SEQ_FEATURE	zinc finger region:C2H2-type 13	2.8	2.45E-04	2.3	3.82E-01	3.82E-01	4.19E-01
	SP_PIR_KEYWORDS	ribosomal protein	3.1	2.54E-04	2.2	1.07E-01	3.71E-02	3.59E-01
	GOTERM_CC_FAT	organelle envelope	6.8	3.25E-04	1.6	1.28E-01	1.94E-02	4.56E-01
	GOTERM_CC_FAT	envelope	6.8	3.41E-04	1.6	1.34E-01	1.78E-02	4.78E-01
	GOTERM_CC_FAT	ribosomal subunit	2.4	3.50E-04	2.5	1.37E-01	1.63E-02	4.91E-01
	GOTERM_CC_FAT	cytosolic ribosome	1.8	4.37E-04	2.9	1.68E-01	1.83E-02	6.12E-01
t	GOTERM_BP_FAT	regulation of transcription, DNA-dependent	12.6	4.69E-04	1.4	6.51E-01	2.96E-01	8.14E-01
	SP_PIR_KEYWORDS	protein biosynthesis	3.1	5.13E-04	2.1	2.04E-01	5.56E-02	7.24E-01
t,z	UP_SEQ_FEATURE	zinc finger region:C2H2-type 15	2.1	5.57E-04	2.6	6.66E-01	4.22E-01	9.52E-01
	GOTERM_BP_FAT	regulation of RNA metabolic process	12.8	5.93E-04	1.4	7.36E-01	2.83E-01	1.03E+00
t,z	UP_SEQ_FEATURE	zinc finger region:C2H2-type 5	5.5	6.24E-04	1.7	7.07E-01	3.36E-01	1.07E+00
t,z	UP_SEQ_FEATURE	domain:KRAB	3.8	6.55E-04	1.9	7.24E-01	2.75E-01	1.12E+00
	SP_PIR_KEYWORDS	ribosome	1.7	6.79E-04	3	2.62E-01	5.88E-02	9.58E-01
t	GOTERM_BP_FAT	regulation of transcription	18.2	6.84E-04	1.3	7.84E-01	2.64E-01	1.18E+00
t,z	UP_SEQ_FEATURE	zinc finger region:C2H2-type 9	4.1	8.16E-04	1.8	7.99E-01	2.75E-01	1.39E+00
t,z	UP_SEQ_FEATURE	zinc finger region:C2H2-type 2; degenerate	1.4	8.81E-04	3.3	8.23E-01	2.51E-01	1.50E+00
	GOTERM_BP_FAT	nucleosome organization	1.4	9.00E-04	3.2	8.67E-01	2.86E-01	1.56E+00
t,z	UP_SEQ_FEATURE	zinc finger region:C2H2-type 12	3.1	9.06E-04	2	8.32E-01	2.25E-01	1.54E+00
	GOTERM_CC_FAT	mitochondrial inner membrane	3.8	9.26E-04	1.9	3.24E-01	3.49E-02	1.29E+00

DOWN IN CHILDREN WITH OLDER FATHERS 2

	Category	Term	%	PValue	Fold	Bonferroni	Benjamini	FDR
	SP_PIR_KEYWORDS	alternative splicing	52.3	4.00E-07	1.3	1.07E-04	1.07E-04	5.26E-04
	UP_SEQ_FEATURE	splice variant	51.7	1.20E-06	1.3	1.24E-03	1.24E-03	1.90E-03
	SP_PIR_KEYWORDS	metal-binding	24.7	2.32E-05	1.5	6.20E-03	2.07E-03	3.05E-02
	SP_PIR_KEYWORDS	zinc-finger	17.2	2.09E-05	1.7	5.58E-03	2.80E-03	2.74E-02
t	GOTERM_BP_FAT	regulation of transcription	21.2	6.68E-06	1.6	1.07E-02	1.07E-02	1.12E-02
	GOTERM_BP_FAT	regulation of RNA metabolic process	15.4	1.35E-05	1.8	2.14E-02	1.08E-02	2.27E-02
t	GOTERM_BP_FAT	regulation of transcription, DNA-dependent	14.9	2.52E-05	1.7	3.96E-02	1.34E-02	4.22E-02
t	SP_PIR_KEYWORDS	transcription regulation	17	5.02E-04	1.5	1.26E-01	2.66E-02	6.57E-01
z	SP_PIR_KEYWORDS	zinc	18.6	4.30E-04	1.5	1.09E-01	2.84E-02	5.63E-01
	SP_PIR_KEYWORDS	serine/threonine-protein kinase	5.3	8.25E-04	2.4	1.98E-01	3.11E-02	1.08E+00
t	SP_PIR_KEYWORDS	Transcription	17.2	8.14E-04	1.5	1.96E-01	3.57E-02	1.06E+00
t	GOTERM_BP_FAT	transcription	17.5	1.26E-04	1.6	1.82E-01	4.91E-02	2.10E-01
t	GOTERM_MF_FAT	DNA binding	18.8	2.85E-04	1.5	1.12E-01	1.12E-01	3.99E-01
z	GOTERM_MF_FAT	zinc ion binding	18.8	9.58E-04	1.4	3.29E-01	1.25E-01	1.34E+00
	GOTERM_MF_FAT	transition metal ion binding	21.8	8.18E-04	1.4	2.89E-01	1.57E-01	1.14E+00
t,z	UP_SEQ_FEATURE	zinc finger region:C2H2-type 5	6.9	3.51E-04	2.2	3.06E-01	1.67E-01	5.56E-01
t,z	UP_SEQ_FEATURE	zinc finger region:C2H2-type 4	6.9	7.94E-04	2.1	5.62E-01	2.41E-01	1.25E+00
	INTERPRO	Serine/threonine protein kinase, active site	5	8.86E-04	2.4	4.05E-01	4.05E-01	1.30E+00

t: transcription-related pathway
z: zinc-related pathway
