# Supplementary material for: Autism and Increased Paternal Age Related Changes in Global Levels of Gene Expression Regulation
Source: PLoS One. 2011 Feb 17;6(2):e16715. doi: 10.1371/journal.pone.0016715 (PMC3040743; doi:10.1371/journal.pone.0016715)
Supplement: Table S2 — Non-overlapping down-regulated genes. Pathway analysis was done on significant gene lists (p<.05, absolute fold change >1.1) of genes that were down-regulated in autism or in children of older fathers and not overlapping with the other group. (PDF) [file pone.0016715.s003.pdf]

Table S2

UP IN AUTISM

Category	Term	%	PValue	Fold	Bonferroni	Benjamini	FDR
UP_SEQ_FEATURE	splice variant	54.63	1.99E-09	1.29	3.05E-06	3.05E-06	3.32E-06
SP_PIR_KEYWORDS	alternative splicing	54.63	2.04E-09	1.29	8.37E-07	8.37E-07	2.86E-06
UP_SEQ_FEATURE	compositionally biased region:Pro-rich	9.62	7.71E-06	1.91	1.17E-02	5.89E-03	1.28E-02
SP_PIR_KEYWORDS	glycoprotein	20.15	1.62E-04	1.39	6.43E-02	3.27E-02	2.27E-01
UP_SEQ_FEATURE	glycosylation site:N-linked (GlcNAc...)	19.06	2.86E-04	1.39	3.55E-01	1.36E-01	4.76E-01
UP_SEQ_FEATURE	compositionally biased region:Gly/Pro-rich	0.91	4.55E-04	12.12	5.02E-01	1.60E-01	7.55E-01
SP_PIR_KEYWORDS	disulfide bond	13.43	4.55E-04	1.49	1.70E-01	6.03E-02	6.35E-01
GOTERM_BP_FAT	GO:0043410~positive regulation of MAPKKK cascade	1.45	6.07E-04	5.28	7.15E-01	7.15E-01	1.04E+00
UP_SEQ_FEATURE	disulfide bond	12.7	8.14E-04	1.48	7.13E-01	2.21E-01	1.35E+00
GOTERM_BP_FAT	GO:0048732~gland development	1.81	1.17E-03	3.76	9.11E-01	7.01E-01	2.00E+00
SP_PIR_KEYWORDS	calcium	5.44	1.30E-03	1.88	4.15E-01	1.25E-01	1.81E+00
SP_PIR_KEYWORDS	signal	15.06	1.50E-03	1.39	4.60E-01	1.16E-01	2.08E+00
UP_SEQ_FEATURE	signal peptide	15.06	1.59E-03	1.39	9.13E-01	3.34E-01	2.61E+00
SP_PIR_KEYWORDS	Secreted	7.26	1.80E-03	1.67	5.22E-01	1.16E-01	2.49E+00
GOTERM_MF_FAT	calcium ion binding	6.17	1.82E-03	1.75	6.99E-01	6.99E-01	2.69E+00
GOTERM_BP_FAT	axon guidance	1.45	3.09E-03	4.05	9.98E-01	8.81E-01	5.20E+00
SP_PIR_KEYWORDS	polymorphism	62.07	5.42E-03	1.09	8.92E-01	2.72E-01	7.32E+00
UP_SEQ_FEATURE	sequence variant	64.43	5.59E-03	1.09	1.00E+00	7.07E-01	8.91E+00
UP_SEQ_FEATURE	compositionally biased region:Arg-rich	2.18	6.36E-03	2.59	1.00E+00	7.05E-01	1.01E+01
GOTERM_BP_FAT	positive regulation of stress-activated protein kinase signaling	0.91	6.41E-03	6.41	1.00E+00	9.64E-01	1.05E+01
GOTERM_BP_FAT	epidermis morphogenesis	0.73	6.51E-03	9.68	1.00E+00	9.33E-01	1.07E+01
GOTERM_BP_FAT	cell morphogenesis involved in neuron differentiation	2.18	6.78E-03	2.56	1.00E+00	9.04E-01	1.11E+01
GOTERM_BP_FAT	fatty acid metabolic process	2.54	7.78E-03	2.29	1.00E+00	9.00E-01	1.26E+01
GOTERM_BP_FAT	positive regulation of signal transduction	3.45	8.41E-03	1.95	1.00E+00	8.87E-01	1.36E+01
KEGG_PATHWAY	Arachidonic acid metabolism	1.09	9.46E-03	4.45	7.31E-01	7.31E-01	1.06E+01

UP IN OLDER FATHERS

Category	Term	%	PValue	Fold En	Bonferroni	Benjamini	FDR
UP_SEQ_FEATURE	compositionally biased region:Pro-rich	9.95	2.66E-07	1.95	4.74E-04	4.74E-04	4.51E-04
KEGG_PATHWAY	Lysosome	3.22	3.85E-06	3.22	6.32E-04	6.32E-04	0.004674
GOTERM_BP_FAT	membrane invagination	3.83	2.42E-05	2.62	0.056015	0.056015	0.042448
GOTERM_BP_FAT	endocytosis	3.83	2.42E-05	2.62	0.056015	0.056015	0.042448
SP_PIR_KEYWORDS	alternative splicing	50.69	3.74E-05	1.18	0.01685	0.01685	0.053155
GOTERM_CC_FAT	vacuole	4.44	3.83E-05	2.33	0.014403	0.014403	0.052959
UP_SEQ_FEATURE	splice variant	50.54	4.39E-05	1.17	0.0752	0.038335	0.074457
GOTERM_BP_FAT	vesicle-mediated transport	7.35	7.22E-05	1.81	0.158	0.082394	0.126584
GOTERM_CC_FAT	lysosome	3.83	9.70E-05	2.4	0.036114	0.018223	0.134214
GOTERM_CC_FAT	lytic vacuole	3.83	9.70E-05	2.4	0.036114	0.018223	0.134214
SP_PIR_KEYWORDS	membrane	35.53	9.79E-05	1.23	0.043478	0.021981	0.138985
SP_PIR_KEYWORDS	lysosome	2.91	1.94E-04	2.72	0.084384	0.028958	0.275452
SP_PIR_KEYWORDS	phosphoprotein	54.06	2.78E-04	1.14	0.11853	0.031049	0.393971
SP_PIR_KEYWORDS	Endocytosis	1.99	4.44E-04	3.32	0.182709	0.039549	0.629286
GOTERM_BP_FAT	vacuole organization	1.38	0.001392	4.01	0.963775	0.66912	2.414171
GOTERM_CC_FAT	extrinsic to membrane	5.51	0.00189	1.71	0.511721	0.212551	2.583569
GOTERM_BP_FAT	membrane organization	4.75	0.001986	1.8	0.991212	0.693824	3.426895
GOTERM_CC_FAT	vesicle	6.58	0.002	1.61	0.531779	0.172796	2.732654
GOTERM_BP_FAT	cholesterol metabolic process	1.68	0.002408	3.11	0.996793	0.682881	4.141221
GOTERM_CC_FAT	cytoplasmic vesicle	6.28	0.002909	1.6	0.66848	0.198134	3.951144
GOTERM_BP_FAT	response to extracellular stimulus	2.76	0.003715	2.16	0.999859	0.771859	6.321916
GOTERM_MF_FAT	vitamin binding	1.84	0.004031	2.72	0.957676	0.957676	5.994894
GOTERM_MF_FAT	identical protein binding	6.43	0.005794	1.53	0.989433	0.897205	8.510684
GOTERM_CC_FAT	internal side of plasma membrane	3.68	0.005804	1.83	0.889881	0.307674	7.739619
UP_SEQ_FEATURE	compositionally biased region:Pro/Ser-rich	0.77	0.005816	6.49	0.999969	0.968606	9.419323
UP_SEQ_FEATURE	repeat:4	1.68	0.006353	2.74	0.999988	0.941354	10.24538
GOTERM_BP_FAT	sterol metabolic process	1.68	0.006768	2.7	1	0.900818	11.23143
GOTERM_BP_FAT	response to nutrient levels	2.45	0.007519	2.13	1	0.894313	12.40215
UP_SEQ_FEATURE	glycosylation site:N-linked (GlcNAc...)	17.3	0.008274	1.25	1	0.948065	13.14262
GOTERM_BP_FAT	leukocyte activation during immune response	1.07	0.008446	3.83	1	0.894047	13.82577
GOTERM_BP_FAT	cell activation during immune response	1.07	0.008446	3.83	1	0.894047	13.82577
GOTERM_MF_FAT	carboxylic acid binding	1.84	0.008819	2.46	0.999028	0.900946	12.68048
GOTERM_BP_FAT	regulation of vesicle-mediated transport	1.53	0.008884	2.78	1	0.880652	14.49233
UP_SEQ_FEATURE	Fe2OG dioxygenase	0.77	0.009689	5.68	1	0.944332	15.22035
