# Supplementary material for: Autism and Increased Paternal Age Related Changes in Global Levels of Gene Expression Regulation
Source: PLoS One. 2011 Feb 17;6(2):e16715. doi: 10.1371/journal.pone.0016715 (PMC3040743; doi:10.1371/journal.pone.0016715)
Supplement: Table S4 — Pathway analysis with increased stringency for gene list selection. Pathway analysis was done on significant gene lists (p<.05, absolute fold change >1.25, and present calls >51%) of genes that were down-regulated in autism and in children of older fathers. Table shows that the same pathways were enriched as with lower stringency gene list selection. (PDF) [file pone.0016715.s005.pdf]

Table S4

DOWN IN BOTH AUTISM AND OLDER FATHERS (high stringency: present in 51% and fold-change > 1.25)

	Category	Term	%	PValue	Fold	Bonferroni	Benjamini	FDR
	SP_PIR_KEYWORDS	ubl conjugation pathway	12.9	3.09E-03	3.99	2.60E-01	2.60E-01	3.37E+00
	GOTERM_BP_FAT	cellular protein catabolic process	14.52	4.35E-03	3.27	9.30E-01	9.30E-01	6.25E+00
	GOTERM_BP_FAT	proteolysis involved in cellular protein catabolic process	14.52	4.35E-03	3.27	9.30E-01	9.30E-01	6.25E+00
	GOTERM_BP_FAT	protein catabolic process	14.52	5.20E-03	3.18	9.60E-01	7.90E-01	7.42E+00
	GOTERM_BP_FAT	modification-dependent macromolecule catabolic process	12.9	1.26E-02	3.03	1.00E+00	9.20E-01	1.72E+01
	GOTERM_BP_FAT	modification-dependent protein catabolic process	12.9	1.26E-02	3.03	1.00E+00	9.20E-01	1.72E+01
	GOTERM_BP_FAT	cellular macromolecule catabolic process	14.52	1.38E-02	2.69	1.00E+00	8.80E-01	1.85E+01
	GOTERM_BP_FAT	macromolecule catabolic process	14.52	1.87E-02	2.55	1.00E+00	9.00E-01	2.43E+01
	GOTERM_BP_FAT	proteolysis	14.52	2.09E-02	2.49	1.00E+00	8.80E-01	2.69E+01
	UP_SEQ_FEATURE	Phosphocysteine intermediate	4.84	2.35E-02	12.32	1.00E+00	1.00E+00	2.66E+01
	INTERPRO	Dual-specific/protein-tyrosine phosphatase, conserved region	4.84	2.40E-02	12.16	9.70E-01	9.70E-01	2.49E+01
	INTERPRO	Protein-tyrosine phosphatase	4.84	2.40E-02	12.16	9.70E-01	9.70E-01	2.49E+01
	INTERPRO	Protein-tyrosine phosphatase, active site	4.84	2.83E-02	11.13	9.80E-01	8.60E-01	2.87E+01
	SP_PIR_KEYWORDS	alternative splicing	45.16	2.87E-02	1.36	9.40E-01	7.60E-01	2.76E+01
	UP_SEQ_FEATURE	splice variant	45.16	2.90E-02	1.36	1.00E+00	9.80E-01	3.18E+01
	GOTERM_CC_FAT	nucleoplasm part	11.29	3.01E-02	2.82	9.70E-01	9.70E-01	2.96E+01
	GOTERM_BP_FAT	intracellular receptor-mediated signaling pathway	4.84	3.43E-02	9.99	1.00E+00	9.50E-01	4.03E+01
	GOTERM_BP_FAT	positive regulation of nucleobase, nucleoside, nucleotide and nucleic	9.68	4.10E-02	3.05	1.00E+00	9.60E-01	4.61E+01
	GOTERM_MF_FAT	protein tyrosine phosphatase activity	4.84	4.53E-02	8.57	1.00E+00	1.00E+00	4.23E+01
t	GOTERM_BP_FAT	regulation of transcription	22.58	4.76E-02	1.68	1.00E+00	9.60E-01	5.14E+01
	GOTERM_BP_FAT	positive regulation of nitrogen compound metabolic process	9.68	4.79E-02	2.92	1.00E+00	9.50E-01	5.16E+01
t	SP_PIR_KEYWORDS	transcription regulation	17.74	5.16E-02	1.89	9.90E-01	8.30E-01	4.44E+01
	GOTERM_BP_FAT	positive regulation of macromolecule metabolic process	11.29	6.30E-02	2.38	1.00E+00	9.70E-01	6.18E+01
t	SP_PIR_KEYWORDS	Transcription	17.74	6.30E-02	1.82	1.00E+00	8.00E-01	5.14E+01
	SP_PIR_KEYWORDS	chromosomal rearrangement	6.45	6.47E-02	4.23	1.00E+00	7.30E-01	5.23E+01
	SP_PIR_KEYWORDS	protein phosphatase	4.84	6.49E-02	7.05	1.00E+00	6.70E-01	5.24E+01
	GOTERM_BP_FAT	regulation of alpha-beta T cell proliferation	3.23	6.51E-02	29.07	1.00E+00	9.70E-01	6.31E+01
	GOTERM_BP_FAT	stem cell maintenance	3.23	6.51E-02	29.07	1.00E+00	9.70E-01	6.31E+01
	GOTERM_BP_FAT	stem cell development	3.23	7.08E-02	26.65	1.00E+00	9.70E-01	6.63E+01
	GOTERM_MF_FAT	transcription cofactor activity	8.06	7.39E-02	3.05	1.00E+00	1.00E+00	5.97E+01
	GOTERM_MF_FAT	transcription activator activity	8.06	7.64E-02	3.01	1.00E+00	9.80E-01	6.10E+01
	GOTERM_BP_FAT	stem cell differentiation	3.23	7.65E-02	24.6	1.00E+00	9.70E-01	6.92E+01
	GOTERM_BP_FAT	protein amino acid dephosphorylation	4.84	7.79E-02	6.31	1.00E+00	9.60E-01	6.99E+01
	UP_SEQ_FEATURE	short sequence motif:LXXLL motif 2	3.23	7.83E-02	24.09	1.00E+00	1.00E+00	6.54E+01
	UP_SEQ_FEATURE	short sequence motif:LXXLL motif 1	3.23	7.83E-02	24.09	1.00E+00	1.00E+00	6.54E+01
	GOTERM_MF_FAT	transcription coactivator activity	6.45	7.93E-02	3.85	1.00E+00	9.50E-01	6.24E+01
	INTERPRO	Armadillo-like helical	4.84	8.52E-02	6.01	1.00E+00	9.80E-01	6.50E+01
t	GOTERM_BP_FAT	positive regulation of transcription	8.06	9.11E-02	2.83	1.00E+00	9.70E-01	7.57E+01
	UP_SEQ_FEATURE	domain:t-SNARE coiled-coil homology	3.23	9.32E-02	20.08	1.00E+00	1.00E+00	7.20E+01
	GOTERM_BP_FAT	intracellular protein transport	8.06	9.48E-02	2.8	1.00E+00	9.70E-01	7.71E+01
	SP_PIR_KEYWORDS	phosphoprotein	48.39	9.63E-02	1.22	1.00E+00	7.60E-01	6.74E+01
t	GOTERM_BP_FAT	positive regulation of gene expression	8.06	9.76E-02	2.77	1.00E+00	9.70E-01	7.81E+01

t transcription-related pathway
